# Influence of sarcopenia on postoperative complications in patients undergoing autologous microsurgical breast reconstruction: an inverse probability of treatment weighting analysis

**DOI:** 10.3389/fonc.2023.1211593

**Published:** 2023-11-02

**Authors:** Seung-Jun Lee, Yun-Jung Yang, Dong-Won Lee, Seung-Yong Song, Dae-Hyun Lew, Eun-Jung Yang

**Affiliations:** ^1^ Department of Plastic and Reconstructive Surgery, Institute for Innovation in Digital Healthcare, Yonsei University College of Medicine, Seoul, Republic of Korea; ^2^ Department of Convergence Science, College of Medicine, Catholic Kwandong University International St. Mary’s Hospital, Incheon, Republic of Korea

**Keywords:** sarcopenia, microsurgery, complication, breast reconstruction, microsurgical breast reconstruction

## Abstract

**Background:**

Sarcopenia is characterized by the loss of skeletal muscle mass and power. Preoperative sarcopenia may be associated with an increased risk of postoperative complications after autologous free-flap breast reconstruction surgery; however, this relationship is controversial.

**Objectives:**

This study aimed to determine whether preoperative sarcopenia is associated with a high complication rate in patients undergoing autologous free-flap breast reconstruction.

**Methods:**

Patients who underwent autologous free-flap breast reconstruction at our hospital between 2019 and 2021 were included in the study. Data on significant complications requiring surgical intervention were retrospectively collected from the medical records. Sarcopenia was defined as having a skeletal muscle index value <41 cm^2^/m^2^. The skeletal muscle index was calculated by dividing the sum of the psoas and iliopsoas muscle areas at the level of the third lumbar vertebra by the patient’s height in meters squared. The relationship between preoperative sarcopenia and postoperative complications was investigated using an inverse probability of treatment weighting (IPTW) analysis.

**Results:**

Among the 203 participants, 90 (44.33%) had preoperative sarcopenia. The general patient characteristics were similar between the sarcopenia and non-sarcopenia groups after IPTW adjustment. Sarcopenia did not significantly increase the risk of flap failure or emergency surgery related to breast reconstruction before IPTW adjustment. However, after IPTW adjustment, the rates of recipient site infection and hematoma were significantly higher in participants with sarcopenia than in those without sarcopenia (*p* < 0.001 and *p* = 0.014, respectively).

**Conclusion:**

Preoperative sarcopenia may influence certain complications of autologous free-flap breast reconstruction surgery.

## 1 Introduction

Breast reconstruction is considered an important factor in recovery from breast cancer surgery, improving the quality of life and mental health of patients. Studies have demonstrated significantly improved quality of life in patients who underwent breast reconstruction compared to that of those who underwent mastectomy alone (1, 2). Despite the drawbacks, such as long operation times and possible complications, autologous tissue free-flap reconstruction is a rational choice for breast reconstruction. However, these disadvantages have lessened over time; appropriate patient selection and improved surgeon skill sets are associated with decreased complication rates (3).

Complications of autologous breast reconstruction include necrosis of the mastectomy flap, infections, hematoma, seroma, and, most importantly, vascular compromise (3). The well-known patient-related factors associated with high complication rates are smoking, diabetes, old age, and overweight (1). Overweight, defined by a body mass index (BMI) >25 kg/m^2^, is a broad metabolic condition with varying presentations. High BMI values can occasionally originate from high muscle mass with adequate or less adipose tissue. Thus, recent reports have focused on the lack of muscle mass, known as sarcopenia, rather than on excessive total body weight (4, 5).

Sarcopenia is defined as an absolute muscle mass below two standard deviations from the mean value observed in healthy young adults (6). Recently, the European Working Group on Sarcopenia for Older People has updated the criteria for defining sarcopenia. These criteria propose screening patients with a suspicion of sarcopenia and confirming the diagnosis *via* muscle mass measurement. However, a global consensus regarding the definition of sarcopenia is lacking (7). Given the widespread use of computed tomography (CT) as an imaging modality, skeletal muscle mass cutoff values calculated by CT imaging are currently used in many clinical settings (7, 8). The prevalence of sarcopenia can range from 3% to 24%, depending on the diagnostic criteria and population age (9). Sarcopenia is also known to be related to inactivity and chronic diseases. The incidence of sarcopenia is increasing, and its association with chronic diseases has been reported (9). Since the association of sarcopenia with poor prognosis in patients with cancer was revealed in 2008 by Prado et al. (10), many follow-up studies have elucidated its clinical significance. Moreover, sarcopenia is also believed to hinder recovery from chemotherapy and surgery (11). Recent studies have revealed the association between complications observed in patients with breast cancer and sarcopenia (12, 13). However, these studies considered various onco-surgical complications instead of those directly related to breast reconstruction alone. Previous research has attempted to prove the importance of sarcopenia in breast reconstruction. Some studies have reported sarcopenia as a discouraging prognostic factor in autologous breast reconstruction; however, some reports have presented contradicting results (4, 5). Therefore, in our study, we attempted to confirm the association between the complications associated with autologous microsurgical breast reconstruction and the patient’s sarcopenia status before surgery. Establishing this connection could lead to more precise patient selection and stratification for autologous microsurgical breast reconstruction surgery, reducing severe complications such as flap detachments. Thus, we aimed to determine the relationship between sarcopenia and high complication rates in autologous microsurgical breast reconstruction.

## 2 Materials and methods

### 2.1 Patients

This study was approved by the research ethics committee (IRB no. 4-2021-1497) of our institution and was conducted in accordance with the Declaration of Helsinki. We retrospectively analyzed the data of 203 patients who underwent surgery, including 12 who underwent bilateral mastectomy. Specimen weight, mastectomy types, and type of free flap were assessed in 215 breasts. Autologous breast tissue reconstructions comprising 188 deep inferior epigastric perforator free flap, 23 transverse rectus abdominis musculocutaneous flap, and four superficial inferior epigastric perforator flap surgeries were performed at our hospital between January 2020 and December 2021. Patients who underwent preoperative chemotherapy and radiotherapy were not excluded. For every patient, mastectomy was performed by breast surgeons, and autologous microsurgical breast reconstruction was subsequently performed by plastic surgeons. The reconstruction surgeries were performed in both an immediate and a delayed manner, respectively. The plastic surgeons included three skilled surgeons, each with more than 10 years of experience. Furthermore, each patient provided informed consent before participating in the study.

### 2.2 Sarcopenia analysis using CT

**Figure 1:**
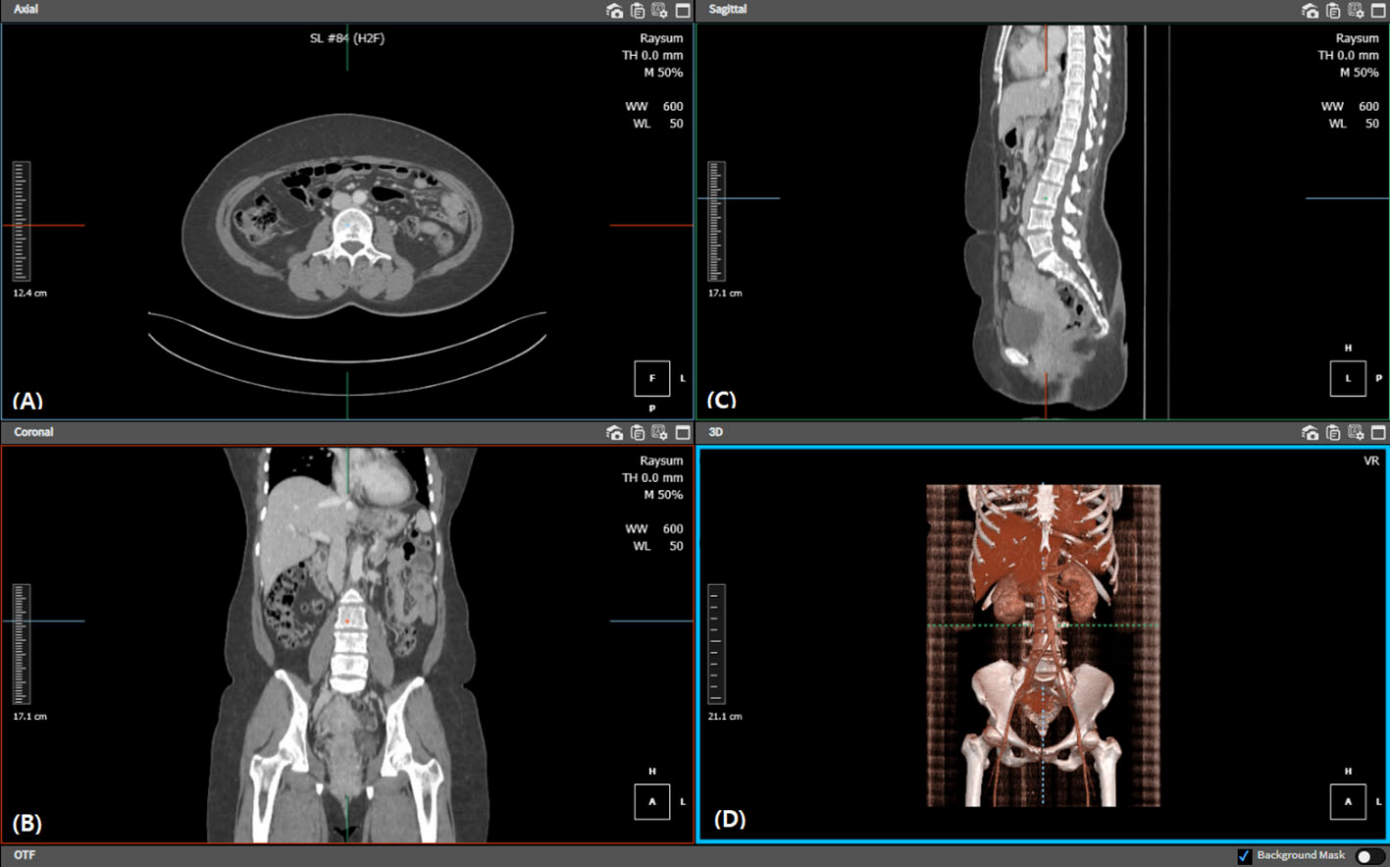
Diagram illustrating the third lumbar level location and calculation of skeletal muscle, subcutaneous fat, and visceral fat areas. Each image shows the procedure of locating the **(A)** third lumbar axial, **(B)** coronal, and **(C)** sagittal planes, respectively. We also confirmed the third lumbar vertebra within the **(D)** 3D reconstructed view. Each tissue filter was applied, and automated calculation was performed on the relevant plane.

**Figure 2:**
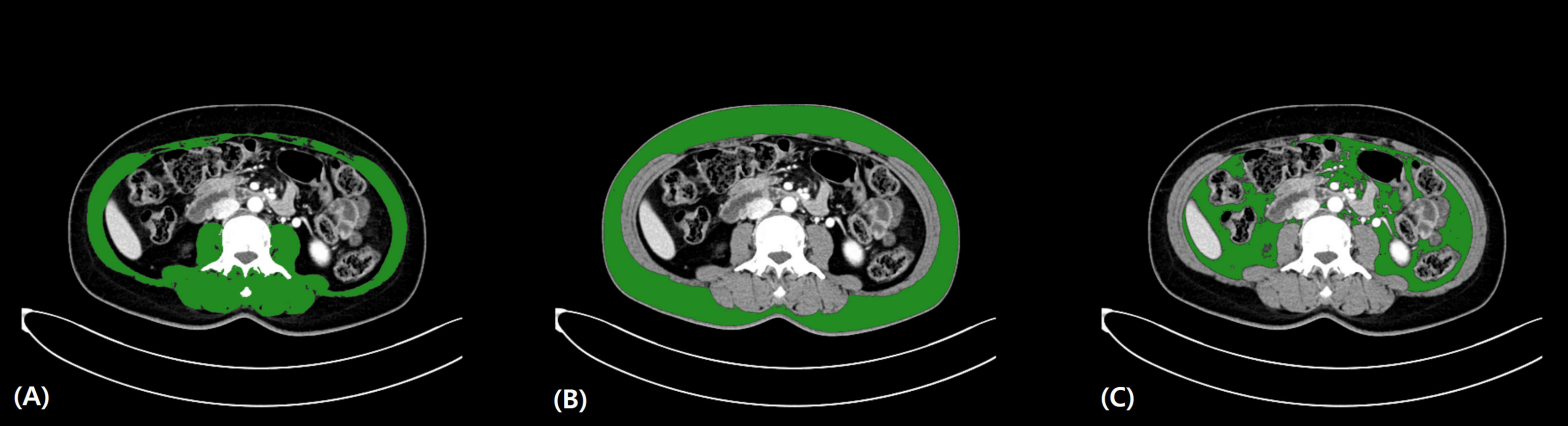
Extracted CT axial cut at the level of the third lumbar vertebra of the **(A)** skeletal muscle, **(B)** subcutaneous fat, and **(C)** visceral fat areas. The HU range was -29 to 150 HU for skeletal muscle tissue, -190 to 30 HU for subcutaneous adipose tissue, and -50 to 150 HU for visceral adipose tissue. CT, computed tomography; HU, Hounsfield unit.

Abdominal pelvic CT was performed for every patient preoperatively. The two-dimensional plane at the level of the third lumbar vertebra was obtained using a tracing method (14) (**Figure 1**), and the area of the skeletal muscle was calculated (-29–150 Hounsfield unit s, HU). The psoas and iliopsoas muscles were selected for muscle area measurement. Next, the sum of these skeletal muscle areas on the third lumbar vertebra plane was divided by the height in meters squared to calculate the skeletal muscle index (SMI). The indices for subcutaneous adipose tissue (-190–30 HU) and visceral adipose tissue (-50–150 HU) were each calculated at the same plane, normalized with height, and were then termed as subcutaneous adipose tissue index and visceral adipose tissue index, respectively. The sum of these values was calculated and termed the total adipose tissue index (**Figure 2**).

The association between the survival rate of patients with breast cancer and sarcopenia defined by SMI <41 cm^2^/m^2^ has been recently confirmed (12–14). Therefore, we defined sarcopenia using the same cutoff value. Patients with values below the cutoff were classified as having sarcopenia (10, 14). The establishment of the criteria for defining sarcopenia is thoroughly discussed in the “Discussion” section.

### 2.3 Outcome assessment

The complications encountered were categorized as perioperative and postoperative. Perioperative complications included total flap loss, mastectomy flap necrosis, emergency arterial thrombosis, emergency venous thrombosis, recipient site infection necessitating a return to the operating room, hematoma requiring a return to the operating room, donor site complication requiring a return to the operating room, and any complication that resulted in a return to the operating room. Postoperative complications included flap fat necrosis, recipient site seroma, infection, dehiscence, and hematoma, and donor site complications. These outcomes were retrospectively recorded and analyzed based on medical records.

Recipient site infection was classified as the presence of either wound discharge, turbid drainage, or admission for intravenous antibiotic therapy. If any procedure was performed in the operating room, including massive irrigation, drain reinsertions, or debridement of infected tissue, the patient was classified as having “recipient site infection leading to a return to the operating room.” Recipient site dehiscence was determined based on whether the patient had undergone a revision procedure. If the procedure was performed at the bedside or in the outpatient department, it was not classified as “recipient site infection leading to a return to the operating room.” Hematoma was determined clinically, such as bruises with palpable masses or sanguineous drainage. When it was removed postoperatively in the operating room, it was classified as a “hematoma leading to a return to the operating room.” Fat necrosis was determined clinically and radiologically. Recipient site seroma was defined as aspiration of seromas. These outcomes were retrospectively analyzed based on medical records.

### 2.4 Statistical analysis

The general and surgical characteristics of each group were described before and after matching for the sarcopenia and non-sarcopenia groups. Baseline characteristics are summarized as means and standard deviations for continuous variables and as counts and proportions for categorical variables. The Shapiro–Wilk test was used to evaluate the normal distribution of continuous variables. Normally distributed continuous variables were then compared using the Student’s *t*-test, whereas skewed data were compared using the Wilcoxon rank-sum test. Categorical variables were compared using the chi-square test or Fisher’s exact test. Univariate and multivariate logistic regression analyses were performed to assess the association between sarcopenia and breast cancer complications. In the multivariate analysis, BMI and specimen weights were included as covariates because they demonstrated significance in the univariate analysis.

To mitigate imbalances in the general and surgical characteristics between groups, inverse probability of treatment weights (IPTW) was applied based on the propensity score (15). The propensity score was estimated using a logistic regression model with the following covariates: age, BMI, hypertension, time of reconstructive surgery (immediate, immediate–delayed, or delayed), and specimen weights. Preoperative radiation was not included as a covariate because these data were only available for the sarcopenia group. After IPTW adjustment, the balance of each covariate was determined.

The rate of postoperative complications was assessed based on 215 breasts because 12 patients underwent bilateral breast reconstruction. Statistical analyses were performed using STATA version 16.1 (StataCorp LP College Station, TX, USA), and statistical significance was set at *p* < 0.05.

## 3 Results

### 3.1 General characteristics before and after IPTW

The general characteristics of the study participants before and after IPTW adjustment are shown in **Table 1**. The study participants were divided into two groups based on their SMI values: <41.0 cm/m^2^ (with sarcopenia) and ≥41.0 cm/m^2^ (without sarcopenia). Before adjustment, 90 of 203 (44.33%) participants met the criteria for sarcopenia. The mean age of the participants without sarcopenia was 47.97 (± 7.99) years and that of the participants with sarcopenia was 49.26 (± 7.56) years. The average BMI of the participants with sarcopenia was significantly lower than that of the participants without sarcopenia (*p* < 0.001). The number of patients with hypertension in the sarcopenia group was significantly lower than the number of patients with hypertension in the non-sarcopenia group (*p* = 0.021), whereas the number of patients who underwent preoperative radiation therapy was significantly higher in the sarcopenia group than that in the non-sarcopenia group (*p* = 0.037). The average age, frequency of smoking, diabetes mellitus, and neoadjuvant chemotherapy were similar between the groups. After IPTW adjustment, the general characteristics, except preoperative radiation therapy, were well balanced between the sarcopenia and non-sarcopenia groups. This is because the patients who underwent preoperative radiation therapy were exclusively in the sarcopenia group.

**Table 1:** General characteristics of the study subjects (*n* = 203) before and after IPTW.

	Number of patients (*n*, %)			P-values	
	All patients (*n* = 203)	Non-sarcopenia (*n* = 113)	Sarcopenia (*n* = 90)	Before adjustment	After adjustment
Age (mean ± SD), years	48.54 ± 7.81	47.97 ± 7.99	49.26 ± 7.56	0.242	0.629
BMI (mean ± SD), kg/m^2^	24.53 ± 3.66	25.94 ± 3.49	22.76 ± 3.06	<0.001	0.812
Smoking				0.194	0.116
Never	195 (96.06)	106 (93.81)	89 (98.89)		
Past smoker	6 (2.96)	5 (4.42)	1 (1.11)		
Current smoker	2 (0.99)	2 (1.77)	0 (0.00)		
Diabetes mellitus	10 (4.93)	6 (5.31)	4 (4.44)	0.522	0.813
Hypertension	23 (11.33)	18 (15.93)	5 (5.56)	0.021	0.182
Preoperative radiation	4 (1.97)	0 (0.00)	4 (4.44)	0.037	0.047
Neoadjuvant chemotherapy	68 (33.50)	36 (31.86)	32 (35.56)	0.579	0.115

Data are shown as mean ± standard deviation and number of patients (percentage). P-values are obtained from the Student’s t-test (or Wilcoxon rank sum test) in continuous variables and chi-square test (or Fisher’s exact test) in categorical variables.

IPTW, inverse probability of treatment weighting; BMI, body mass index; SD, standard deviation.

### 3.2 Surgical characteristics before and after IPTW

Considering the patients who underwent bilateral mastectomy, the type of free flap, time, and mastectomy type were based on the number of breasts (*n* = 215). The number of participants (*n* = 203) was also based on the laterality of mastectomy and hospitalization days. Before IPTW adjustment, the average weight of the breast specimens was significantly lower in the participants with sarcopenia than in those without sarcopenia (*p* = 0.004; **Table 2**). The frequencies of delayed or immediate–delayed breast reconstruction were significantly higher in the participants with sarcopenia than in those without sarcopenia (*p* = 0.037). The number of literalities of mastectomy, including the type of free-flap, mastectomy type, average specimen weight, and the frequencies of delayed or immediate–delayed breast reconstruction and hospitalization days, did not differ between the two groups. After IPTW adjustment, no significant differences were observed in any of the surgical characteristics between the sarcopenia and non-sarcopenia groups.

**Table 2:** Surgical characteristics of the study subjects (*n* = 203) before and after inverse probability of treatment weighting.

	Number of breast (*n*, %)			*P*-values	
	All breasts (*n* = 215)	Non-sarcopenia (*n* = 121)	Sarcopenia (*n* = 94)	Before adjustment	After adjustment
Laterality of mastectomy (*n* = 203)				0.429	0.441
Unilateral	191 (94.09)	105 (92.92)	86 (95.56)		
Bilateral	12 (5.91)	8 (7.08)	4 (4.44)		
Type of free flap				0.818	0.612
DIEP	188 (87.44)	105 (86.78)	83 (88.30)		
TRAM	23 (10.70)	13 (10.74)	10 (10.64)		
SIEA	4 (1.86)	3 (2.48)	1 (1.06)		
Time				0.037	0.556
Immediate	178 (82.79)	107 (88.43)	71 (75.53)		
Immediate–delayed	16 (7.44)	7 (5.79)	9 (9.57)		
Delayed	21 (9.77)	7 (5.79)	14 (14.89)		
Specimen weight (mean ± SD), kg/m^2^	526.52 ± 236.00	560.09 ± 225.06	483.31 ± 243.83	0.004	0.552
Mastectomy type				0.840	0.115
NSM	120 (55.81)	65 (53.72)	55 (58.51)		
SSM	42 (19.53)	25 (20.66)	17 (18.09)		
TM	43 (20.00)	26 (21.49)	17 (18.09)		
MRM	10 (4.65)	5 (4.13)	5 (5.32)		
Hospitalization days (mean ± SD), days (*n* = 203)	10.07 ± 2.34	10.19 ± 2.50	9.93 ± 2.14	0.491	0.418

Data before adjustment are shown as mean ± standard deviations and number of patients/breasts (percentage). P-values are obtained from the Student’s t-test (or Wilcoxon rank sum test) in continuous variables and chi-square test (or Fisher’s exact test) in categorical variables.

DIEP, deep inferior epigastric perforator; TRAM, transverse rectus abdominis myocutaneous; SIEA, superficial inferior epigastric artery; NSM, nipple-sparing mastectomy; SSM, skin-sparing mastectomy; TM, total mastectomy; MRM, modified radical mastectomy; SD, standard deviation.

### 3.3 Association between sarcopenia and overall breast reconstruction-elated complications

The odds ratios (ORs) and 95% confidence intervals (CIs) for breast reconstruction-related complications according to sarcopenia are presented in **Table 3**.

**Table 3:** Association between sarcopenia and breast reconstruction-related postoperative complications (*n* = 215) before and after IPTW.

Analysis		OR (95% CI)	*P*-values
Before adjustment	Crude analysis	0.80 (0.46–1.38)	0.432
	Multivariate analysis	0.92 (0.49–1.74)	0.820
After adjustment	ATE using IPTW	0.95 (0.46–1.96)	0.904

The multivariate analysis was adjusted with body mass index and specimen weights.

ATE, average treatment effect; IPTW, inverse probability of treatment weighting.

Before adjustment, sarcopenia did not significantly increase breast reconstruction-related complications (OR: 0.80, 95% CI: 0.46–1.38; **Table 3**) in the univariate analysis. However, a significant association between BMI, specimen weights, and the risk of breast reconstruction-related complications was observed (OR: 1.07, 95% CI: 1.00–1.15 and OR: 1.002, 95% CI: 1.000–1.003, respectively; **Supplementary Table S1**).

In the multivariate analysis, sarcopenia did not increase the risk of perioperative complications (OR: 0.92, 95% CI: 0.49–1.74). The association between BMI and the risk of breast reconstruction-related complications was not significant in the multivariate analysis (OR: 1.04, 95% CI: 0.91–1.19; **Supplementary Table S1**). However, specimen weights were significantly associated with the risk of breast reconstruction-related complications (OR: 1.002, 95% CI: 1.000–1.003; **Supplementary Table S1**).

After IPTW adjustment, sarcopenia was not associated with the risk of breast reconstruction-related complications (OR: 0.95, 95% CI 0.46–1.96).

### 3.4 Rates of peri- and postoperative complications after breast reconstruction

The prevalence of each postoperative complication was compared between groups (**Table 4**). Among the 215 breasts, 98 (45.58%) met the criteria for postoperative complications of breast reconstruction. Before adjustment, mastectomy flap necrosis was the most common major complication in the sarcopenia and non-sarcopenia groups (25.62% and 14.89%, respectively); however, statistical significance was not detected. The prevalence of emergency arterial and venous thrombosis, recipient site infection, hematoma, and donor site complications was not significantly different between the two groups.

**Table 4:** Comparisons of postoperative complications between sarcopenia and non-sarcopenia groups.

Postoperative complications	Number of breasts (*n*, %)			*P*-values	
	All breasts (*n* = 215)	Non-sarcopenia (*n* = 121)	Sarcopenia (*n* = 94)	Before adjustment	After adjustment
Overall	98 (45.58)	58 (47.93)	40 (42.55)	0.432	0.903
Postoperative complications					
Flap fat necrosis	13 (6.05)	9 (7.44)	4 (4.26)	0.331	0.184
Recipient site seroma	7 (3.26)	5 (4.13)	2 (2.13)	0.472	0.137
Recipient site infection	5 (2.33)	1 (0.83)	4 (4.26)	0.170	<0.001
Recipient site dehiscence	14 (6.51)	7 (5.79)	7 (7.45)	0.476	0.084
Recipient site hematoma	11 (5.14)	6 (5.00)	5 (5.32)	1.000	0.014
Any donor site complication	17 (7.94)	10 (8.33)	7 (7.45)	0.812	0.606
Perioperative major complications					
Total flap loss	14 (6.51)	7 (5.79)	7 (7.45)	0.624	0.900
Mastectomy flap necrosis	45 (20.93)	31 (25.62)	14 (14.89)	0.055	0.455
Emergency arterial thrombosis	5 (2.33)	1 (0.83)	4 (4.26)	0.170	0.144
Emergency venous thrombosis	6 (2.79)	3 (2.48)	3 (3.19)	1.000	0.335
Recipient site infection return to OR	4 (1.86)	1 (0.83)	3 (3.19)	0.321	0.089
Hematoma return to OR	8 (3.72)	4 (3.31)	4 (4.26)	0.732	0.292
Donor‐site complication return to OR	3 (1.40)	2 (1.65)	1 (1.06)	1.000	0.365
Return to OR	52 (24.19)	31 (25.62)	21(21.28)	0.578	0.647

Data are shown as number of breasts (percentage). P-values are obtained from chi-square test (or Fisher’s exact test) in categorical variables.

OR, operating room.

After IPTW adjustment, among minor complications, the rates of recipient site infection and hematoma were significantly higher in the participants with sarcopenia than in those without sarcopenia (*p* < 0.001 and *p* = 0.014, respectively). Perioperative complications, including mastectomy flap necrosis, were not significantly different between the two groups.

## 4 Discussion

We aimed to determine the relevance of sarcopenia in other breast reconstruction-related complications, such as peri- and postoperative complications. Major perioperative complications, such as total flap loss or emergency re-anastomosis, were irrelevant in patients with sarcopenia as indicated by the lack of statistical significance even after IPTW adjustment. In contrast, postoperative complications, such as infections and hematoma, were positively related to sarcopenia after IPTW adjustment.

Sarcopenia results from a multi-pathway pathogenesis and is not solely a consequence of a sedentary lifestyle (9, 16). Chronic inflammation, hormonal changes, reduced protein and vitamin D intakes, and low muscle satellite cell activation contribute to sarcopenia pathogenesis (17)— for instance, well-known conditions associated with it, such as aging and lack of exercise, can be attributed to aging-related inflammation and low muscle cell stimulation (17).

From the comparison of postoperative recipient site infection, we inferred that sarcopenia would adversely affect the patient’s immunity. Infection is usually determined by the patient’s immune system status and adherence to aseptic protocols in the institution. Previous reports have also implicated a relationship between sarcopenia and the immune system. Some studies in oncological surgery have suggested preoperative sarcopenia as a predictive factor for postoperative infection (18, 19). Takano et al. and Yasuhiro et al. reported the relationship between sarcopenia and high postoperative infection in gastric and colorectal cancer surgeries (20, 21). A recent study focused on the relationship between sarcopenia and the systemic immune system; however, the results were inconclusive (22). Our findings further reinforce the uncertain relationship between postoperative infection and sarcopenia.

Postoperative hematoma is another complication that is influenced by the prevalence of sarcopenia. However, unlike infection, studies evaluating the relationship between postoperative hematoma and sarcopenia in cancer surgeries are lacking. Nevertheless, similar reports have stated that preoperative sarcopenia is an independent risk factor for postoperative major organ bleeding in cardiovascular interventions, such as coronary stenting or left ventricular assist device support (23). While the cause remains unclear, the frailty of patients with sarcopenia might lead to a bleeding tendency due to the lack of coagulative components (23, 24). In cardiovascular interventions, sarcopenia is more pronounced and related to the outcome as it can directly affect cardiac mass and function (24), thus displaying a slightly different pathophysiology from that in onco-reconstructive surgeries. Some studies have focused on the relationship between serum coagulation factor levels and sarcopenia. Chen et al. reported that the fibrinogen levels were higher in patients with sarcopenia because of the high inflammatory conditions (25). This finding was not directly related to our results; however, sarcopenia may influence the coagulation pathway and systemic inflammation. Azuma et al. reported that sarcopenia is related to decreased serum vitamin K levels (26), which aligns with our results. These results suggest the presence of definite relationships between the coagulation pathway and sarcopenia. To our knowledge, ours is the first study to propose a positive relationship between postoperative hematoma and sarcopenia after an onco-reconstructive surgery.

In this study, the lumbar SMI based on CT images was used to classify sarcopenia. The SMI cutoff points for sarcopenia have not yet been standardized. The cutoff range for sarcopenia in women, determined using SMI, is 29.6–41 cm^2^/m^2^ (13). The most common cutoff points are 52.4 cm^2^/m^2^ (male individuals) and 38.5 cm^2^/m^2^ (female individuals), which were suggested in a study that confirmed the association between muscle mass and mortality in 250 overweight patients with respiratory or gastrointestinal malignancies in Canada (10). Subsequently, Martin et al. confirmed the optimal threshold for muscle mass loss associated with increased mortality in patients with lung or gastrointestinal malignancies (27). Consequently, the threshold for men was 43 cm^2^/m^2^ for those with BMI <25 and 53 cm^2^/m^2^ for those with BMI >25. Additionally, 41 cm^2^/m^2^ was suggested as an appropriate cutoff point for women regardless of BMI (27). Several recent studies have confirmed the association between sarcopenia and the survival rates of patients with breast cancer based on SMI <41 cm^2^/m^2^ (28–30). Therefore, in this study, SMI of 41 cm^2^/m^2^ was used as the cutoff point to classify participants with sarcopenia. Considering that the appropriate threshold for sarcopenia in patients with breast cancer remains unknown, the cutoff point used in this study (41 cm^2^/m^2^) may have resulted in overestimation or underestimation of sarcopenia. As such, our study proposed the first SMI cutoff value for sarcopenia definition in microsurgical breast reconstruction.

The patient factors that could serve as risk factors for breast reconstruction-related complications are presented in **Supplementary Table S1**. Being overweight is a well-known risk factor for complications in autologous microsurgical reconstruction (31). Before IPTW adjustment, the BMI in the group without sarcopenia was higher than that in the group with sarcopenia. Thus, to establish a causal relationship between sarcopenia and complications, we deemed it necessary to adjust for BMI. After IPTW adjustment, the groups exhibited no significant difference in BMI, which allowed us to assess the risk of complications directly associated with sarcopenia. The multivariate analysis revealed that no specific patient factors, including BMI, were related to breast reconstruction-related complications, contrary to the results of a previous preliminary report (31). BMI and sarcopenia are often considered simultaneously, as both factors are related to body composition. We confirmed that sarcopenia can affect the perioperative complication rate. Furthermore, we suggested that, compared to BMI, sarcopenia might be a more sensitive predictor of microsurgical risk in patients with cancer. Further studies with a larger number of patients under various conditions are warranted to confirm this.

In addition, neoadjuvant chemotherapy is known to contribute to the development of sarcopenia and is also associated with poor prognosis during treatment (32). The proportion of patients who received neoadjuvant chemotherapy was not significantly different between the two groups (34.04 vs. 36.84) in the univariate analysis.

We conducted a comprehensive analysis of various factors related to breast reconstruction outcomes. However, a limitation regarding the statistical analyses employed should be acknowledged. We performed numerous statistical comparisons without applying adjustments for multiple testing. While providing insights into potential associations, this approach increases the likelihood of type I errors. Therefore, we recognize the need for caution in interpreting the statistical significance of individual results. To address this limitation, future research in this area may benefit from employing appropriate multiple testing corrections to ensure the robustness and reliability of findings. Furthermore, considering the low incidence of complications, the sample size was not large enough. Consequently, the low number of complications in each category might have limited the establishment of causality. Moreover, no widely accepted and established definition of sarcopenia is available; therefore, different definitions can yield conflicting results. Nonetheless, this study underscores the importance of considering sarcopenia in patients undergoing microsurgical breast reconstruction. When microsurgical breast reconstruction is planned for patients with sarcopenia, which can be easily determined by preoperative CT, lifestyle modification attempts and balanced nutrition intake, along with detailed risk assessment and information, are recommended.

## 5 Conclusion

In conclusion, preoperative sarcopenia is an independent risk factor for postoperative complications of autologous tissue breast reconstruction, such as postoperative infection and hematoma. The cause remains unknown, but frailty due to sarcopenic conditions, including decreased major organ function, might be crucial. Therefore, extra caution should be exercised when performing autologous microsurgical breast reconstruction in patients with sarcopenia.

## Data availability statement

The original contributions presented in the study are included in the article/**Supplementary Material**. Further inquiries can be directed to the corresponding author.

## Ethics statement

The studies involving humans were approved by the Severance Hospital Research Ethics Committee. The studies were conducted in accordance with the local legislation and institutional requirements. The human samples used in this study were acquired from primarily isolated data as part of a previous study for which ethical approval was obtained. Written informed consent for participation was not required from the participants or the participants’ legal guardians/next of kin in accordance with the national legislation and institutional requirements.

## Author contributions

S-JL and E-JY have made substantial contributions to the conception and design or data acquisition. D-WL, S-YS, and D-HL have made substantial contributions to data acquisition. Y-JY was involved in the analysis and interpretation of data. S-JL and E-JY were involved in drafting the manuscript or revising it critically for important intellectual content. All authors contributed to the article and approved the submitted version.
