# Supplementary material for: Influence of sarcopenia on postoperative complications in patients undergoing autologous microsurgical breast reconstruction: an inverse probability of treatment weighting analysis
Source: Front Oncol. 2023 Nov 2;13:1211593. doi: 10.3389/fonc.2023.1211593 (PMC10652871; doi:10.3389/fonc.2023.1211593)
Supplement: Supplementary file 1 [file Table_1.docx]

Supplement Table 1. The association between independent risk factors and breast reconstruction-related complications

|  | **Univariate analysis** | | **Multivariate analysis** | |  |
| --- | --- | --- | --- | --- | --- |
|  | **OR (95% CI)** | **p-value** | **OR (95% CI)** | **p-value** |  |
| Age | 1.00 (0.97-1.04) | 0.710 |  |  |  |
| BMI | 1.07 (1.00-1.15) | 0.044 | 0.99 (0.90-1.10) | 0.938 |  |
| Smoking history |  |  |  |  |  |
| Never | Ref |  |  |  |  |
| Past smoker | 2.50 (0.44-13.95) | 0.296 |  |  |  |
| Current smoker | - |  |  |  |  |
| Diabetes mellitus | 0.99 (0.29-3.36) | 0.993 |  |  |  |
| Hypertension | 0.66 (0.29-1.53) | 0.343 |  |  |  |
| Preoperative radiation | 0.79 (0.12-4.83) | 0.800 |  |  |  |
| Neoadjuvant chemotherapy | 0.76 (0.43-1.35) | 0.360 |  |  |  |
| Laterality of mastectomy | |  |  |  | |
| Unilateral | Ref |  |  |  |  |
| Bilateral | 0.56 (0.22-1.37) | 0.206 |  |  |  |
| Type of free flap |  |  |  |  |  |
| DIEP | Ref |  |  |  |  |
| TRAM | 0.95 (0.39-2.28) | 0.913 |  |  |  |
| Other free flap | - | - |  |  |  |
| Time |  |  |  |  |  |
| Immediate | Ref |  |  |  |  |
| Immediate-delayed | 0.91 (0.32-2.55) | 0.859 |  |  |  |
| Delayed | 0.68 (0.25-1.81) | 0.445 |  |  |  |
| Specimen weight (mean ± SD), kg | 1.002 (1.000-1.003) | 0.001 | 1.002 (1.000-1.003) | 0.007 |  |
| Mastectomy type |  |  |  |  |  |
| NSM | Ref |  |  |  |  |
| SSM | 0.65 (0.32-1.34) | 0.253 |  |  |  |
| TM | 0.76 (0.38-1.55) | 0.466 |  |  |  |
| MRM | 1.60 (0.43-5.97) | 0.482 |  |  |  |

BMI: body mass index; DIEP: deep inferior epigastric perforator; TRAM: transverse rectus abdominis myocutaneous; NSM: nipple sparing mastectomy; SSM: skin sparing mastectomy; TM: total mastectomy; MRM: modified radical mastectomy; SD: standard deviation
